# Dexmedetomidine had neuroprotective effects on hippocampal neuronal cells via targeting lncRNA SHNG16 mediated microRNA-10b-5p/BDNF axis

**DOI:** 10.1007/s11010-020-03726-6

**Published:** 2020-04-22

**Authors:** Li Wang, Weihua Liu, Yanjun Zhang, Zhanfei Hu, Hao Guo, Jingshu Lv, Hongyin Du

**Affiliations:** 1 Clinical College of the First Center of Tianjin Medical University, Tianjin, China; 2 Department of Anesthesiology, Tianjin First Center Hospital, No. 24 Fukang Road, Nankai District, Tianjin, 300192 China

**Keywords:** Dexmedetomidine, SHNG16, miR-10b-5p, BDNF, Neuroprotection

## Abstract

Dexmedetomidine (DEX), a highly selective alpha2 adrenergic receptor agonist, is a commonly used anesthetic drug in surgical procedures. Previous studies have indicated that DEX exerts neuroprotective effects while the detailed mechanism has not been fully elucidated. Here, we aim to study the role of lncRNA SHNG16 in DEX-induced brain protection and its underlying molecular mechanism. The rats underwent middle cerebral artery occlusion (MCAO) surgery and oxygen–glucose deprivation (OGD)-treated HT22 hippocampal neurons were treated with DEX, respectively. CCK8 was used to evaluate cell viability. sh-SHNG16 as well as miR-10b-5p mimics were transfected into hippocampal neurons to further explore the bio-function of SNHG16 and miR-10b-5p in vitro. Furthermore, the interactions between SHNG16 and miR-10b-5p, miR-10b-5p and BDNF gene were confirmed by dual-luciferase report assay. Our data revealed that DEX attenuated neurological damage of the MCAO rats and also increased the cell viability of the neurons significantly. Besides, expression of SHNG16 and BDNF were both downregulated while miR-10b-5p was upregulated in MCAO brain tissues or OGD treated neurons. DEX inhibited miR-10b-5p expression but increased SHNG16 and BDNF levels with a dosage effect. After transfection with sh-SHNG16 or miR-10b-5p mimics, the expression of BDNF protein was downregulated, accompanied with decreased neuron viability. Dual-luciferase assay showed that SHNG16 targeted on miR-10b-5p, which also could bind directly to the 3′-UTR sites of BDNF and negatively regulate its expression. In conclusion, DEX exerts neuroprotective in ischemic stroke via improving neuron damage, the underlying mechanism may be upregulating SHNG16 and BDNF via sponging miR-10b-5p.

## Introduction

Ischemic cerebrovascular disease remains one of the diseases with the highest morbidity, disability, and mortality in the world, which has also been a serious threat to the health and quality of life of the middle-aged and elderly people [1]. From the perspective of the pathogenesis involving ischemic injury, cerebral blood supply disorder is a crucial factor leading to ischemia, hypoxia, and focal ischemic necrosis of brain tissues. Currently, thrombolysis and other treatment methods are adopted to restore the local blood supply. However, reperfusion itself can lead to excitatory amino acid toxicity, apoptosis, intracellular calcium overload and other reperfusion injuries [2–4]. Therefore, it is of great significance to explore new effective therapeutic methods against ischemic/reperfusion induced injury.

Dexmedetomidine (DEX), a new highly selective alpha2 adrenergic receptor agonist, has been found to have pharmacological properties, such as analgesia, inhibition of sympathetic activity with a dose-dependent effect but without respiratory depression [5]. In recent years, a large number of in vivo and in vitro studies have shown that DEX can exert neuroprotective effects through a variety of mechanisms. For example, DEX can increase the expression of brain-derived neurotrophic factor (BDNF) in astroglia cells through ERK-dependent pathway, thereby diminishing neuronal death caused by glutamate agonists [6]. Additionally, DEX can also reduce the neurotoxicity of neonatal rats mediated by cerebral ischemia–reperfusion by weakening the TLR4/NF-κB signaling pathway [7]. However, the role and mechanism of DEX in ischemic brain injury need further research.

Long non-coding RNA (lncRNA) is a non-coding RNA with a length of more than 200 nucleotides. LncRNAs are involved in a wide range of biological and cellular processes through regulating genetic expression in epigenetic, transcriptional, or post-transcriptional level [8, 9]. Previous studies have shown that lncRNAs play an important role in neural development, such as regulating the differentiation of neural stem cells into neurons, glial cells, and astrocytes. Meanwhile, abnormal expression of lncRNAs is also closely related to neurological diseases [10]. SNHG16 is a member of lncRNA, and previous research indicates that it exerts significant effect in regulating a variety of tumors, such as pancreatic cancer and gastric cancer [11, 12]. However, the effect of SNHG16 in neuronal cell damage has not been clarified.

Similar to lncRNAs, microRNAs are a class of small intracellular molecules and also belong to non-coding RNAs (about 22 nucleotides in length). After transcription, microRNAs interact with the complementary sequences of their targeted mRNAs in the 3′-UTR sites in the posttranscription level, thus regulating their expression by promoting the degradation of mRNA or inhibiting mRNA translation [13]. Studies have found that miRNA has a prominent role in regulating nerve injury and protection. For example, miR-204 may modulate the pathological injury process of hypoxic-ischemic encephalopathy and the proliferation and apoptosis of neurons by targeting gene killin p53 regulated DNA replication inhibitor (KLLN), which can inhibit DNA synthesis and promote S phase arrest coupled to apoptosis [14], while miR-26b was found to regulate the inflammatory response of microglia cells during hypoxia/ischemia and affect the development of vascular cognitive impairment [15]. MiR-10b-5p, a vital member of miRNAs, has been found to play a significant role in colon cancer, glioma, and other malignant tumors [16, 17], but the mechanism of DEX mediated neuroprotective effect remains to be further explored.

Brain-derived neurotrophic factor (BDNF) has been proved to be a key regulator in neurite growth, synaptic plasticity, and functional neuronal connection selection in the central nervous system, and is one of several endogenous proteins that play a key role in the survival, maintenance and growth of brain and peripheral neurons [18]. In previous studies, we found that neurons were significantly injured and the expression of BDNF was significantly decreased in the hypoxic and ischemic environment. However, under the effects of DEX, the proliferation ability of neurons was enhanced and apoptosis was weakened, accompanied with the upregulation of SNHG16 and BDNF. In addition, our bioinformatics analysis showed that miR-10b-5p is an important target of SNH16 and also targeted BDNF. In conclusion, this study explored the protective role of DEX in ischemic brain injury and its function in regulating the SNHG16/miR-10b-5p/BDNF axis.

## Materials and methods

### Animals and drug treatments

The modified Zea-Longa suture method was conducted to establish a focal ischemia model of the left middle cerebral artery in rats [19]. After 2 h of focal ischemia of the middle cerebral artery, the suture plug was removed to the external carotid artery to carry out reperfusion for another 24 h, then the model of ischemia–reperfusion was completed. In the sham group, the rats didn’t receive ligation or insertion and only the common carotid artery, internal carotid artery/external carotid artery and vagus nerve were separated. 5-point method was used to evaluate neurologic functions after 2 h of ischemia. 0 score: no neurological defect; 1 point: lift tail contralateral trunk or forelimb bending, or do not extend the front claw when hanging vertically; 2 points: crawling hovering to the opposite side, but normal rest posture; 3 points: loss of correct reflex; 4 points: no spontaneous physical activity. The rats with 1–3 points were successfully modeled and rats with 0 and 4 points were eliminated. In the treatment group, DEX (25 μmol/kg, 50 μmol/kg, 100 μmol/kg body weight) was given the external jugular vein injection at the beginning of reperfusion. The sham group received the same dose of solvent.

### Culture and treatment of HT22 cells

HT22 mouse hippocampal neuronal cell line was purchased from the American Type Culture Collection (ATCC, Manassas, VA, USA) and cultured in DMEM high glucose medium containing 10% fetal bovine serum (FBS) (GIBCO, Shanghai, China). The medium was cultured in a humidified incubator containing 5% CO_2_ at 37 °C, and the medium was changed every 48 h. After the monolayer cultured cells were confluent, the cells were digested with 0.25% trypsin, and the cells in the logarithmic growth phase were subjected to further experiments. HT22 neuronal cells were treated with different concentrations of DEX (25 μM, 50 μM, 100 μM) for 24 h. Cell viability (CCK8 analysis) and LDH levels were measured immediately after 24 h of DEX exposure.

### Establishment of an in vitro oxygen–glucose deprivation/reoxygenation (OGD/R) model

An in vitro ischemic brain injury model was established using the oxygen–glucose deprivation/reoxygenation (OGD/R) model. In brief, HT22 neuronal cells were washed 3 times with glucose-free DMEM and transferred to an incubator containing 1% O_2_, 5% CO_2_, and 94% N_2_ for 1.5 h at 37℃. The culture medium is then replaced with the normal complete culture medium, and the petri dish is put back into the normal incubator. The control group was cultured in the nerve base medium in the 5% CO_2_ atmosphere incubator for the same time.

### Cell transfection

GenePharma (Shanghai, China) synthesized miR-10b-5p mimics, miR-10b-5p inhibitors, and corresponding NC mimic and NC inhibitor. The short hairpin RNAs (shRNAs) for SNHG16 were connected to the PGPU6/Neo plasmid (Genepharma) to silence the expression of SNHG16, which is designated as sh-SNHG16. The empty vector shScramble was used as a blank control. Cell transfection was performed for 48 h in a 6-well plate (5 × 10^5^ cells/well) with Lipofectamine 3000 reagent (Life Technologies Corporation, Carlsbad, CA, USA).

### Measurement of cell viability

HT22 cells in logarithmic growth stage were trypsinize with trypsin and the cell density was adjusted to 2 × 10^3^/mL. The cells were inoculated in 96-well plates with 100-micron cell suspension per well and 3 replicates in each group. After that, the 96-well plate was placed in the incubator for further cultivation. After 24 h of DEX treatment, 10 μL CCK8 (Beyotime Biotechnology, Shanghai, China) was added to each well when the cell growth was stable and then incubated in the incubator for 1 h. After termination of culturing, the 96-well plate was placed in the microplate reader to measure the absorbance (OD value) of each well at 450 nm wavelength. After that, the cell absorbance was measured at the 24th hour.

### Quantitative polymerase chain reaction (qPCR) assay

Total RNA was extracted from cells using TRIzol reagent (Invitrogen, USA) according to the manufacturer's protocol. The purity and concentration of RNA were determined with UV spectrophotometer. According to the manufacturer's instructions, RNA is reverted to cDNA using the PrimescriptTM RT reagent kit with gDNA Eraser (TaKaRa, Japan). SYBR Green PCR Master Mix (TaKaRa, Ohtsu, Japan) was used for qPCR reaction. The relative gene expression was calculated by 2^−ΔΔct^ method, BDNF and SNHG16 had GAPDH as an internal reference, and miRNA had U6-snRNA as an internal reference. The PCR primers of BDNF were: forward primer: 5′-GGGACCGGTTTGTGT-3′, reverse primer: 5′-TTGCTTTTTCATGGGGGCA-3′; SNHG16 forward, 5′-ATGCAGGTTCCGTCTCAGAA-3′, reverse primer: 5′-ACGCCTTTCCATGATGCTTC-3′; forward primer: 5′-GACAGCCGCATCTTCT-3′, reverse prime: 5′-GCGCCCAATACGACCAAATC-3′; miR-10b-5p forward primer: 5′-CAGCAGCACACTGTGGTTTGTA-3′, reverse primer was universal primers (Uni-miR qPCR Primer), U6-snRNA, forward primer: 5′-CTCGCTTCGGCAGCACA-3′, reverse primer: 5′-ACGCTTCACGAATTTGCGT-3′.

### Dual-luciferase reporter assay

The cDNA sequences of wild-type (WT) BDNF and SNHG16 3′-UTR were cloned into pGL3 vectors (Promega, Madison, MI, USA) to establish a dual-luciferase reporter vector BDNF-WT and SNHG16-WT. To produce mutated 3′-URT, the mutation was generated using QuikChange II XL Site‐Directed Mutagenesis Kit (Stratagene). The mutated BDNF and SNHG16 3′-UTR sequences were also cloned into pGL3 vectors to create another dual-luciferase reporter vector, BDNF-Mut, and SNHG16-Mut. Besides, the synthetic miR-10b-5p mimics and non-specific NC mimics were purchased from GenePharma (Shanghai GenePharma, Shanghai, China). HT22 cells were seeded in 48-well plates (2 × 10^3^ cells per well). After 24 h of incubation, the cells were conducted transfection using lipofectamine 2000 (Invitrogen) according to the manufacturer's protocol. After 48 h, the Dual-Luciferase Reporter Assay System (Promega) was used to measure the firefly luciferase activity. All experiments were performed in triplicate and repeated three times independently.

### Western blot

Total protein was extracted from cultured cells or fresh frozen rat brain tissues. The protein concentration was determined by BCA protein assay (Beyotime Institute of Biotechnology, China). Proteins from each sample were separated by SDS-PAGE and then transferred electrochemically to PVDF membranes (Millipore, USA) for immunoblot analysis. Rabbit anti-BDNF (1:1000, ab108319; Abcam) primary antibody was incubated with the membranes at 4 °C on a shaking table. After that, the membranes were incubated with horseradish peroxidase-conjugated goat anti-rabbit IgG (1:5000, BL003A; Biosharp, St. Louis, MO, USA) for another 2 h at room temperature. Then the enhanced chemiluminescence for visualization (Thermo Scientific, Waltham, MA) was used to test the strips, which were analyzed by ImageJ software. GAPDH was used as an internal reference for BDNF.

### Enzyme-linked immunosorbent assay (ELISA)

The expression level of BDNF in the cell culture medium was measured by ELISA. The cell culture medium was taken, and the cells and cell debris were removed by centrifugation. The experiment was performed according to the instructions of the BDNF ELISA kit, and the BDNF concentration of the medium was calculated. All experiments were performed in triplicate and repeated three times independently.

### Immunofluorescence staining

HT22 cells were fixed by 4% paraformaldehyde for 10 min at 24 h after different treatments. Next, PBS was used to wash the cells for 3 times and 10 min each time. Then the cells were incubated with BDNF primary antibody (Abcam, ab108319, 1:200) overnight at 4 °C. Then cells were rinsed by PBS three times and incubated with goat anti-rabbit antibodies (BA1105; Boster, Wuhan, China, 1:200) for 1 h at the 37 °C using water-bath heating. 4,6-diamidino-2-phenylindole (DAPI, C1006, Beyotime, China) was used to stain the nuclei. Immunoreactive proteins were visualized and imaged by a fluorescence microscope (Olympus BX53, Nikon, Japan).

### Statistical analysis

The data were expressed as mean ± SD of at least three independent experiments for each cell group. One-way analysis of variance (ANOVA) followed by the post hoc Duncan test was adopted for all statistical analyses. GraphPad Prism (version 5; GraphPad Software, Inc., La Jolla, CA) was used for analysis. *P* < 0.05 was considered to be statistically significant.

## Results

### DEX attenuated neuronal cell damage in cerebral ischemia

**Fig. 1:**
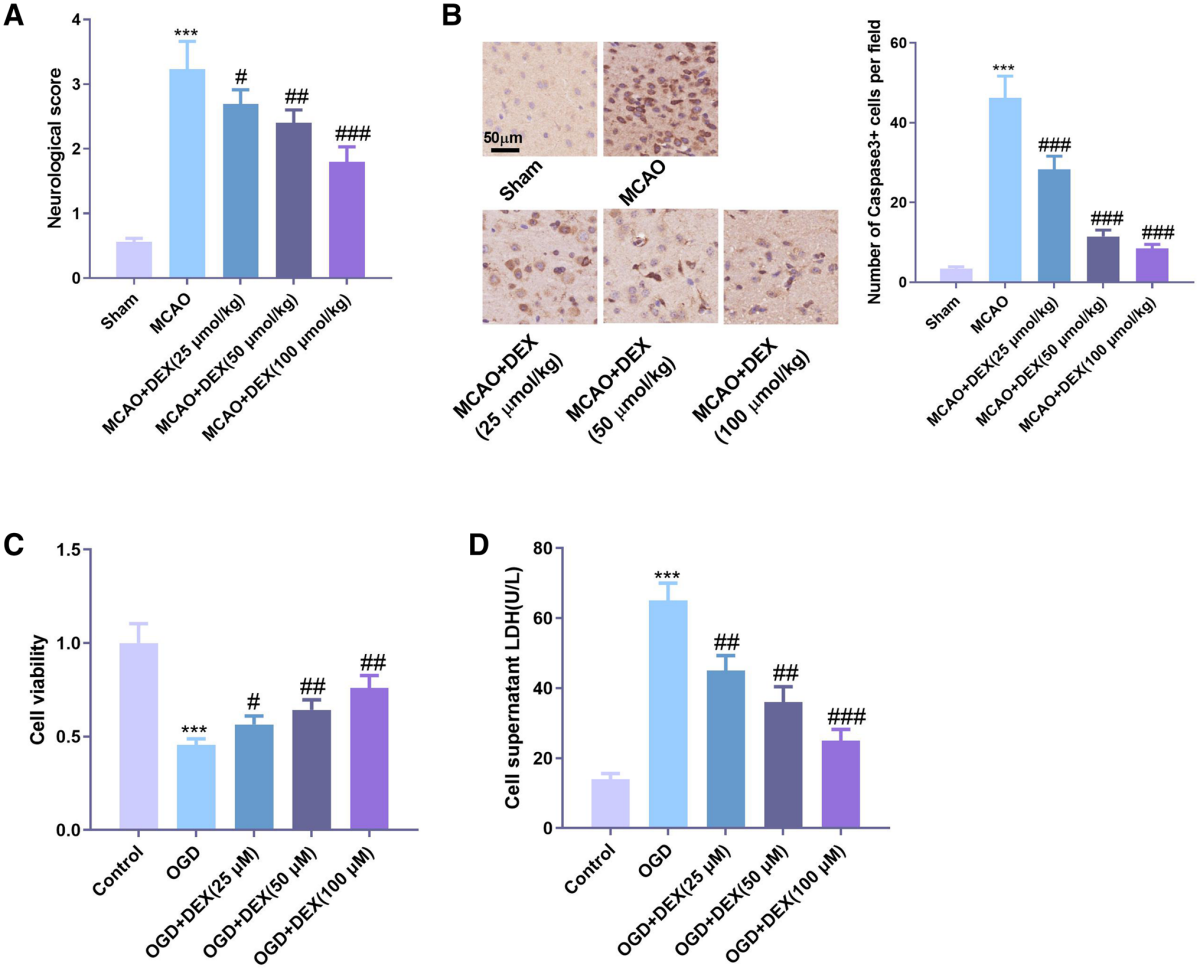
DEX attenuated neuronal cell damage in cerebral ischemia. Rat models of MCAO-induced ischemic brain injury were established and different concentrations of DEX were administered to the rats. **a** Assessment of neurological function in rats 3 days after MCAO. **b** Detection of Caspase-3-labeled apoptotic neurons by tissue immunochemistry; establishment of oxygen–glucose deprivation (OGD) cell model. **c** Detection of cell viability using CCK8 assay. **d** The LDH content in the supernatant culture medium was measured using the LDH kit. *** indicates *P* < 0.001 compared with the Sham group or Control group; ^#^, ^##^, ^###^ indicates *P* < 0.05, *P* < 0.01 and *P* < 0.001 compared with MCAO group or OGD group

To preliminarily explore the protective effects of DEX on neuronal injury, we established a rat model of cerebral ischemia and then treated rats with different concentrations of DEX. Evaluation of neurological function in rats showed that DEX significantly promoted the recovery of neurological function in ischemic rats (Fig. 1a). Immunohistochemistry of Caspase-3 was used to detect the apoptosis of neurons in the brain, and the results showed that cerebral ischemia significantly increased the number of Caspase-3 labeled neurons, while DEX notably inhibited the number of Caspase-3 positive cells, and the inhibitory effect was more remarkable with the increase of DEX concentration (Fig. 1b). In addition, OGD/R method was used to establish the model of cerebral ischemia in vitro. The results showed that OGD/R considerably reduced the cell viability of HT22 cells (Fig. 1c) and increased the expression of LDH in cells (Fig. 1d). However, under the action of DEX, the cell viability of HT22 cells increased, while the release of LDH decreased drastically with a dose-dependent effect.

### DEX upregulated the expression of SNHG16 and BDNF and inhibited the expression of miR-10b-5p

**Fig. 2:**
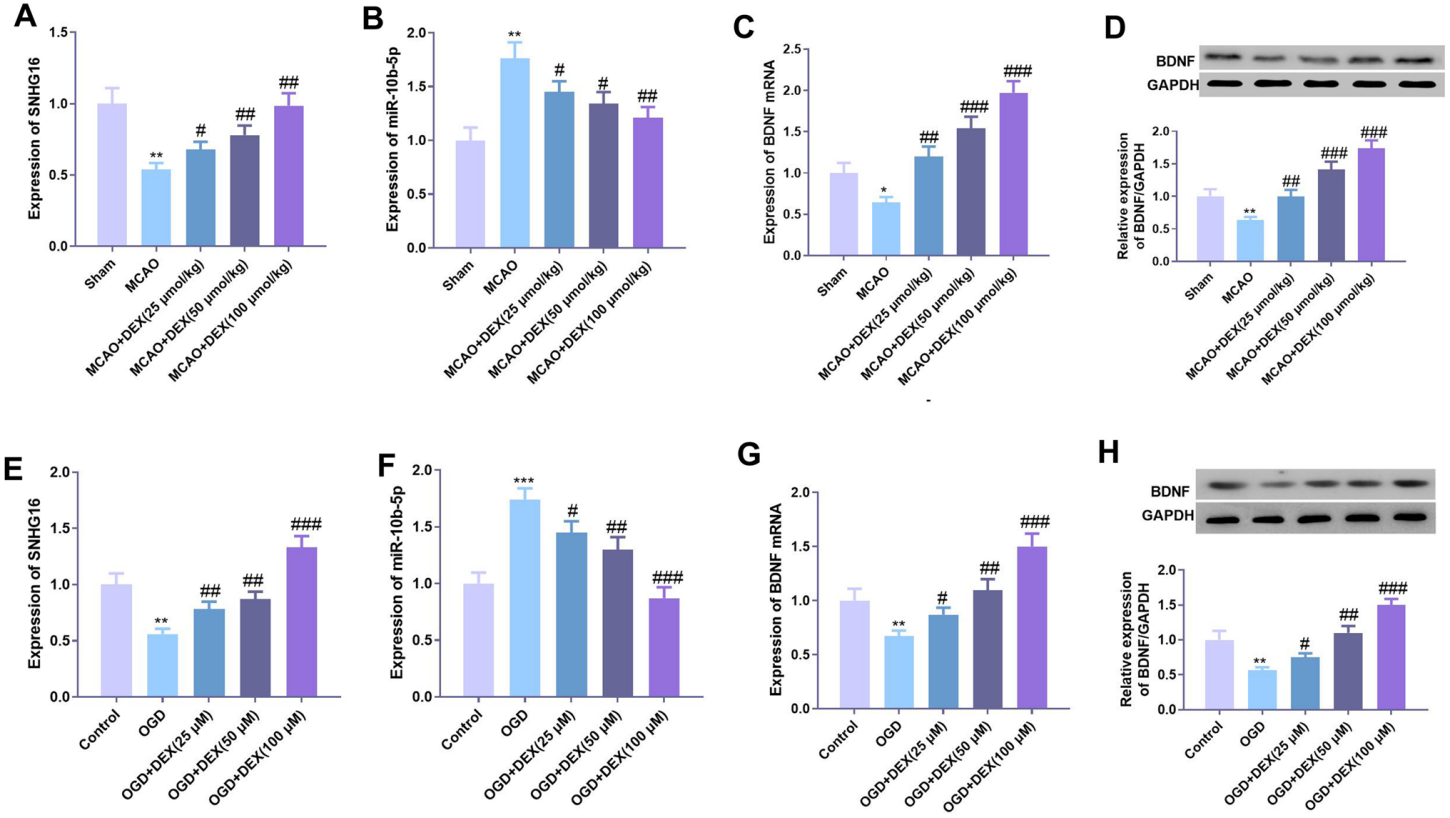
DEX upregulated the expression of SNHG16 and BDNF, and inhibited the expression of miR-10b-5p. The expression of SNHG16 (**a**), miR-10b-5p (**b**) and BDNF mRNA (**c**) in the brain tissues of MCAO rats was detected by qPCR after DEX treatment at different concentrations, and the protein expression of BDNF in the tissues was detected by Western blot (**d**). An in vitro OGD neuron injury model was established. After DEX treatment at different concentrations, the expression of SNHG16 (**e**), miR-10b-5p (**f**) and BDNF mRNA (**g**) in neurons was detected by qPCR, and the protein expression of BDNF in neurons was detected by Western blot (**h**). *, **, *** represents *P* < 0.05, *P* < 0.01 and *P* < 0.001 compared with the Sham group or Control group. ^#^, ^##^, ^###^ indicates that compared with MCAO group or OGD group, *P* < 0.05, *P* < 0.01 and *P* < 0.001

To further explore the specific mechanism of the protective effect of DEX on neurons, we used qPCR to detect the expression of SNHG16, miR-10b-5p, and BDNF in brain tissue and cells. The results showed that cerebral ischemia considerably inhibited the expression of SNHG16 and BDNF but promoted the expression of miR-10b-5p compared with the control group (Fig. 2a–c) in the rat model. Under the treatment of DEX, the expression of SNHG16 and BDNF was significantly upregulated, and the expression of miR-10b-5p was remarkably downregulated (Fig. 2a–c). Similarly, at the cellular level, DEX also promoted the expression of SNHG16 and BDNF and inhibited the expression of miR-10b-5p (Fig. 2d–h).

### Low expression of SNHG16 promoted hypoxia-induced neuronal injury

**Fig. 3:**
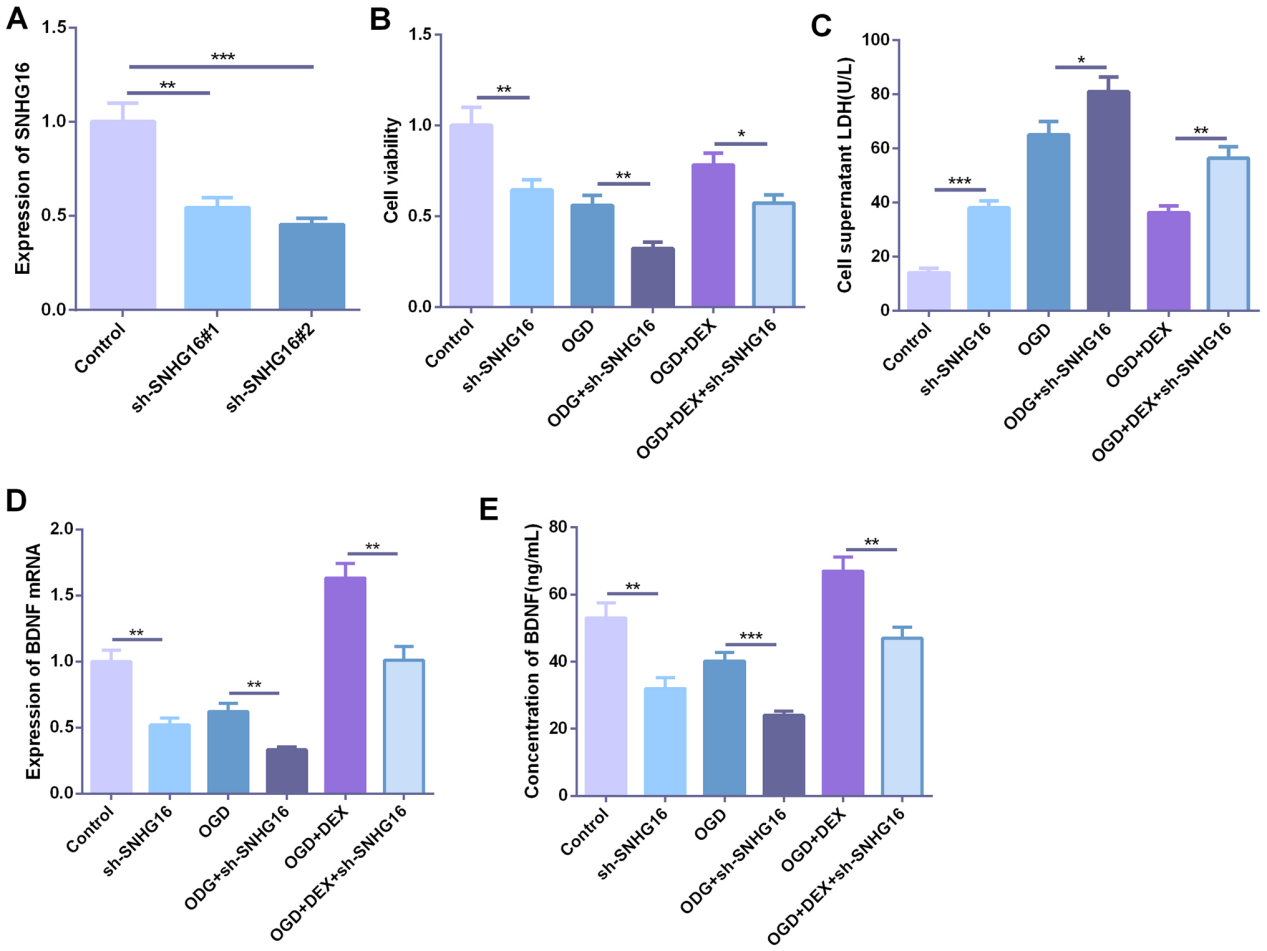
Low expression of SNHG16 promoted hypoxia-induced neuronal injury. **a** ShRNA was used to construct a low expression of SNHG16 cell model with two parallel duplicated samples. Basing on this, the ODG cell model was established and DEX was applied to the cells. **b** The cell viability was measured by the CCK8 method. **c** LDH content in the supernatant culture medium was detected by the LDH kit. **d**, **e** The expression of BDNF in cells and culture medium was detected by qPCR and ELISA, respectively. *,**,** represents *P* < 0.05, *P* < 0.01 and *P* < 0.001, respectively

To further explore the role of SNHG16 in hypoxia-induced neuronal injury, we established a cell model with low expression of SNHG16 (Fig. 3a). Further establishment of oxygen–glucose deprivation (OGD) cell model revealed that the viability of the cells was significantly decreased after knocking down SNHG16, and the LDH content produced by the cells was significantly increased (Fig. 3b, c). After DEX treatment, the cell viability was increased, but knocking down SNHG16 further inhibited the cell viability of neurons (Fig. 3b, c). The expression of BDNF in cells and culture medium was detected by qPCR and ELISA, respectively. The results showed that the expression of BNDF was significantly decreased after knockdown of SNHG16, and the expression of BNDF was further decreased under the environment of OGD. DEX promoted the expression of BDNF, but the expression of BNDF was considerably attenuated after knocking down SNHG16 (Fig. 3d, e).

### SNHG16 targeted miR-10b-5p

**Fig. 4:**
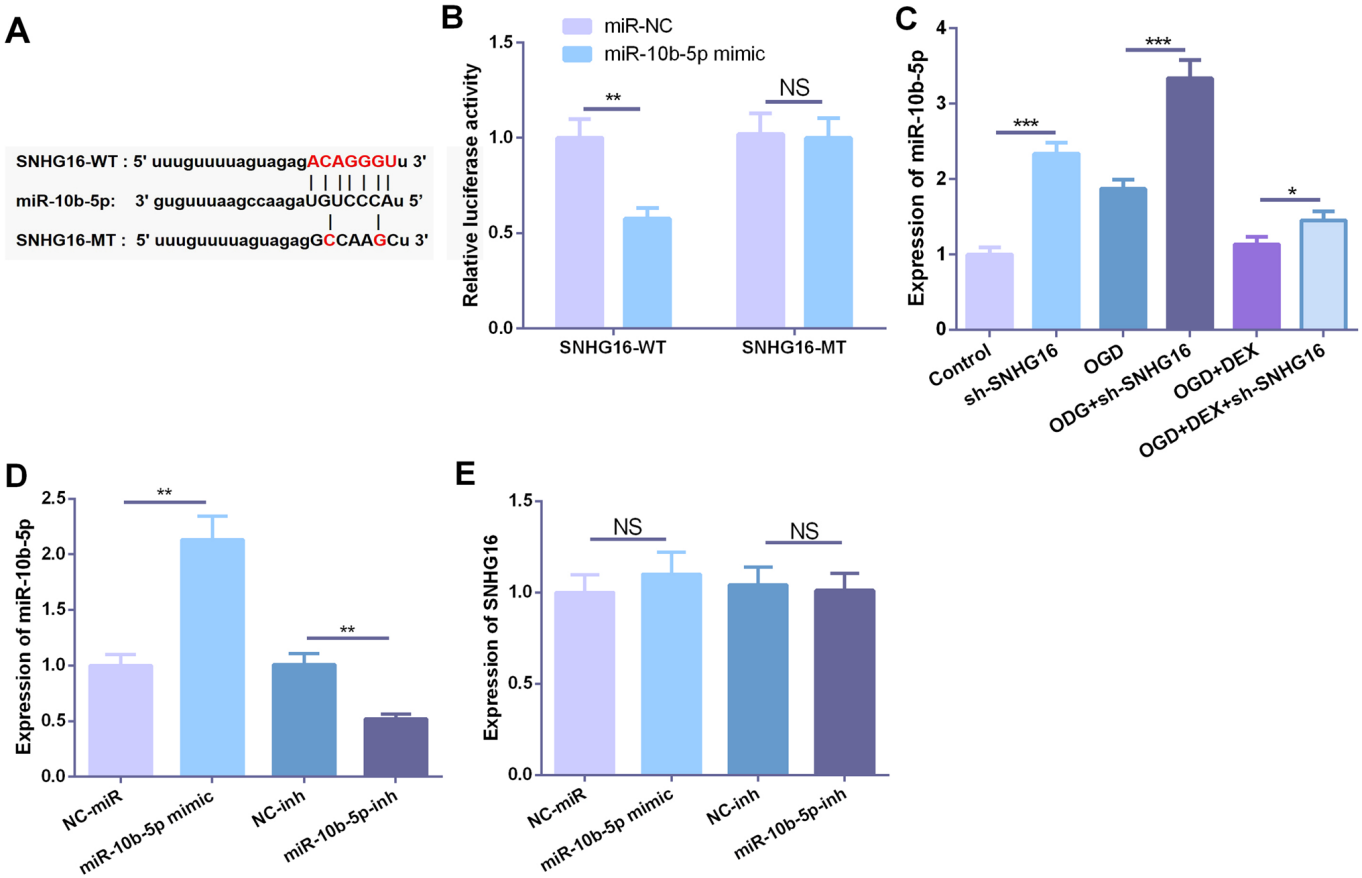
SNHG16 targeted miR-10b-5p. **a** Using StarBase (https://starbase.sysu.edu.cn) to predict the downstream miRNA of SNHG16 and the result showed that miR-10b-5p was an important target. **b** The dual-luciferase activity assay was used to verify the targeting relationship between SNHG16 and miR-10b-5p. **c** Detection of miR-10b-5p expression by qPCR. **d** Construction of miR-10b-5p overexpression and low expression cell model. **e** Detection of SHNG16 expression by qPCR. NS, *, **, *** represents *P* > 0.05, *P* < 0.05, *P* < 0.01 and *P* < 0.001, respectively

In recent years, more and more studies have confirmed that lncRNA can function as competitive endogenous RNA (ceRNA) in regulating miRNA expression and function. In previous studies, we performed a predictive analysis to explore the downstream miRNAs of lncRNA through StarBase (https://starbase.sysu.edu.cn), which showed that SNHG16 can specifically bind to the putative target sequence of miR-10b-5p (Fig. 4a). To further verify the targeting relationship between the two molecules, we performed dual-luciferase reporter assay, which showed that miR-10b-5p can inhibit the luciferase activity of SNHG16-WT, but had no significant effect on the luciferase activity of SNHG16-MT (Fig. 4b). Besides, the expression of miR-10b-5p was detected by qPCR and it was found that the expression of miR-10b-5p was significantly upregulated after OGD insult or knockdown of SNHG16, whereas DEX inhibited the expression of miR-10b-5p (Fig. 4c). To further explore the role of miR-10b-5p in the regulation of SNHG16 expression, we constructed a low expression and overexpression cell model of miR-10b-5p (Fig. 4d). qPCR results indicated that miR-10b-5p had no significant effect on SNHG16 expression (Fig. 4e).

### BDNF was targetedly inhibited by miR-10b-5p

**Fig. 5:**
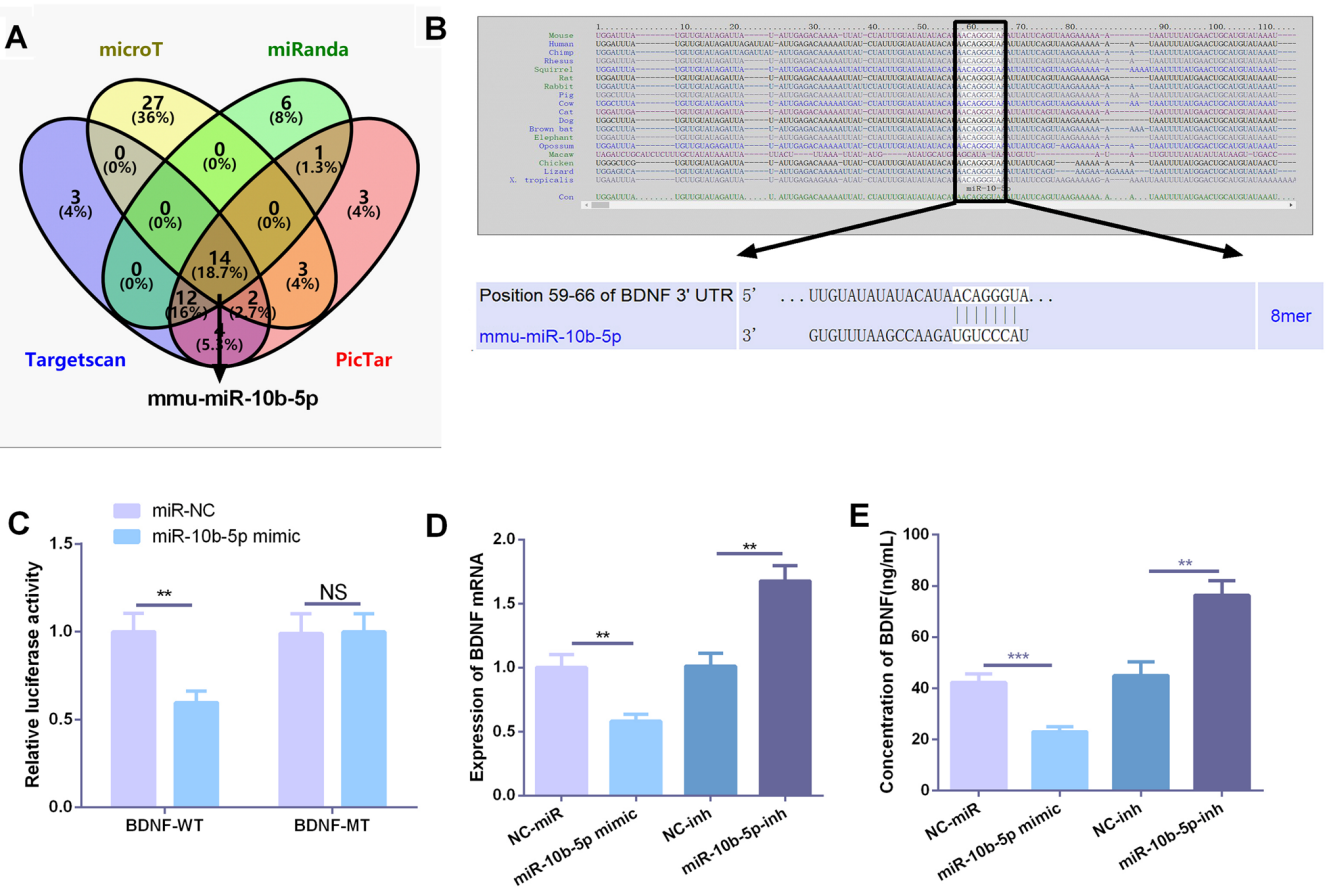
BDNF was targeted and inhibited by miR-10b-5p. **a** Targeted regulatory molecules of BNDF were analyzed online by Targetscan, microT, miRanda, and PicTar databases, and the jointly predicted molecules were analyzed by the Venn diagram, of which miR-10b-5p was an important member. **b** Site schematic diagram of miR-10b-5p targeting BDNF. **c** The targeting relationship between BDNF and miR-10b-5p was verified by dual-luciferase activity assay. **d**, **e** Based on the cell models of overexpression and downexpression of miR-10b-5p, the expression of BDNF in cells and medium was detected by qPCR and ELISA, respectively. **,*** represents *P* < 0.01 and *P* < 0.001, respectively

In order to further explore the upstream and downstream regulatory relationship of BDNF, we analyzed the targeted regulatory miRNAs of BNDF through Targetscan, microT, miRanda, and PicTar databases online. It was found that miR-10b-5p was one potential molecule of them (Fig. 5a, b). Therefore, we are greatly interested in the targeted regulatory effect of miR-10b-5p on BDNF. The dual-luciferase activity experiment was performed, and the results showed that miR-10b-5p mimic significantly reduced the luciferase activity of BDNF-WT, but had no obvious effect on the luciferase activity of BDNF MT (Fig. 5c), suggesting that BDNF was a target of miR-10b-5p. Besides, results of qPCR and ELISA showed that overexpression of miR-10b-5p significantly inhibited BDNF expression, while knockdown of miR-10b-5p promoted BDNF expression (Fig. 5d, e). These results fully indicated that miR-10b-5p could target regulate the expression of BDNF.

### SNHG16 regulates OGD-mediated neuronal injury through miR-10b-5p

**Fig. 6:**
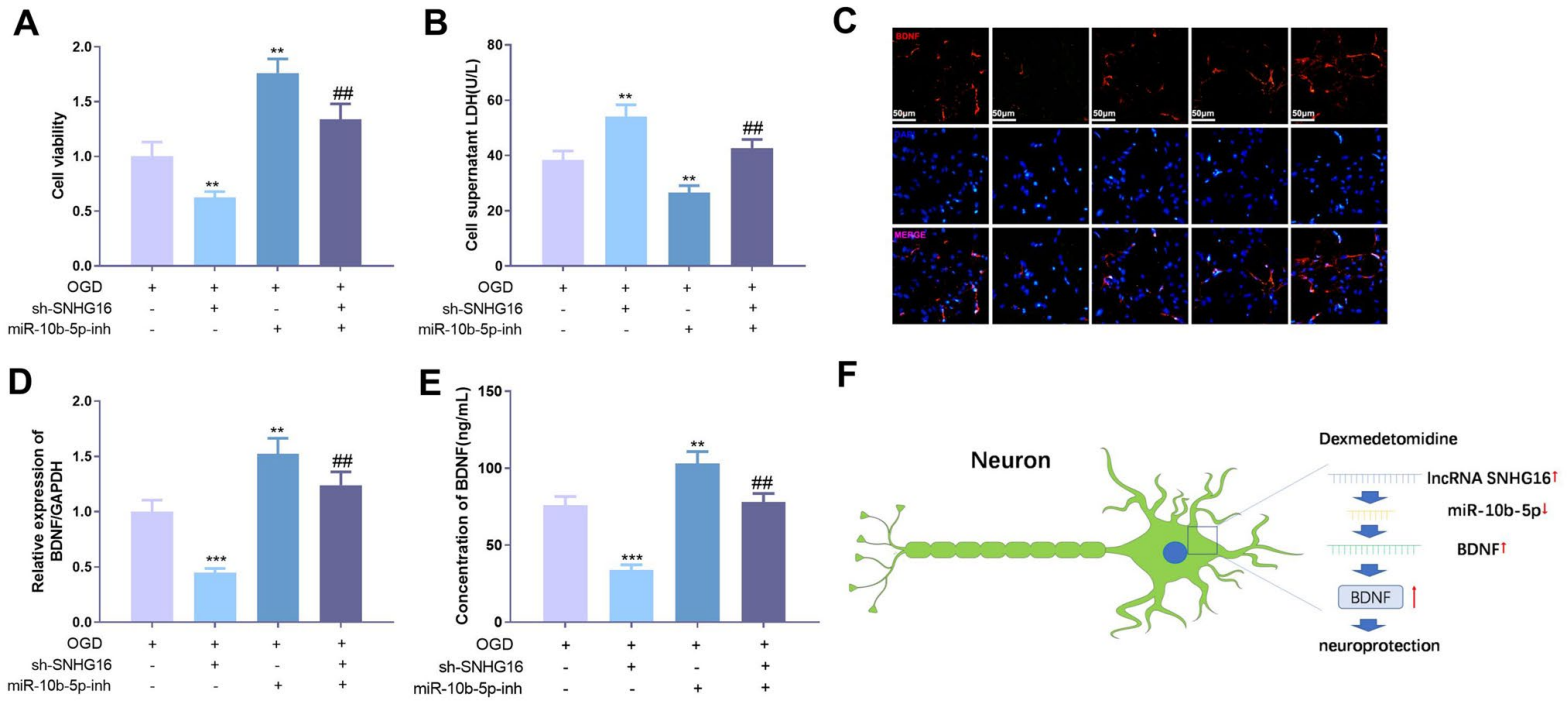
SNHG16 regulated OGD-mediated neuronal damage via miR-10b-5p. A low expression cell model of SNHG16 was established, on the basis of low expression of miR-10b-5p by its specific inhibitor. **a** The cell proliferation ability was measured by the CCK8 method. **b** The LDH content in the medium was measured using an LDH kit. **c** Immunofluorescence staining was used to detect BNDF expression in HT22 cells. Scale bar = 100 µm. **d**, **e** The expression of BDNF in cells and culture medium was detected by qPCR and ELISA, respectively. **f** A diagrammatic sketch map of DEX induced neuroprotective effects on HT22 neurons under OGD treatment. **, *** represents *P* < 0.05, *P* < 0.01 and *P* < 0.001 compared with OGD group, ^##^ represents *P* < 0.01 compared with the sh-SNHG16 group

To explore the role of SNHG16 and miR-10b-5p in OGD-mediated neuronal damage, we downregulated the expression of miR-10b-5p using miR-10b-5p inhibitor on the basis of knockdown of SNHG16. Further examination of cell viability and LDH content revealed a significant increase in cell viability after downregulation of miR-10b-5p compared to knockdown of the SNHG16 group (Fig. 6a), while LDH content was significantly decreased (Fig. 6b). Detection of BDNF expression revealed that knockdown of SNHG16 remarkably downregulated BDNF expression, whereas BDNF expression was significantly upregulated after inhibition of miR-10b-5p (Fig. 6c–e). These results indicated that SNHG16 can promote the expression of BDNF by inhibiting the expression of miR-10b-5p.

## Discussion

In recent years, accumulating evidence show that dexmedetomidine can have a significant neuroprotective effect on nervous system diseases. For example, dexmedetomidine can significantly reduce the nerve injury caused by hyperoxia in newborn rats [20], and also notably reduce the apoptosis of neurons connected by ischemia–reperfusion [21]. Additionally, dexmedetomidine was also found to enhance protective effects of glutamate on the nerve injury-induced by isoflurane in newborn rats [22]. Presently, this research further explored the protective effect and mechanism of dexmedetomidine on ischemic hypoxia-induced nerve injury through in vitro and in vivo experiments. Our results confirm that dexmedetomidine significantly attenuates hypoxia-induced apoptosis of neurons.

As a new class of non-coding RNA molecules, lncRNA has been found to play an important role in the progression of neurological diseases in recent years. For example, lncRNA BACE1-AS is upregulated in Alzheimer's disease (AD) brain tissue and it stabilizes BACE1 mRNA and promotes BACE1 protein expression and Aβ42 formation [23]. In Hirschsprung's disease (HSCR), apoptotic neurons can inhibit apoptosis in non-apoptotic cells by secreting extracellular bodies containing high levels of HN12-lncRNA [24]. In chronic compressive injury (CCI), lncRNA MRAK009713 is a major regulator of neuropathic pain in rats and is significantly increased in CCI rats with enhanced pain behavior [25]. Interestingly, abnormal expression of various lncRNAs makes much sense in ischemic brain injury. For example, LncRNA MEG3 [26], lncRNA TUG1 [27], etc. are significantly upregulated in ischemic brain tissue and can mediate neuronal apoptosis, whereas lncRNA-N1LR enhances ischemic stroke by inhibiting p53 phosphorylation neuroprotective effects [28]. Similarly, SNHG16 is also a member of the lncRNA molecule, and several studies have indicated that it can promote the progression of malignant tumors. Furthermore, SNHG16 reverses LPS-induced inflammatory response in neurosepsis [29] and also attenuates hydrogen peroxide-induced PC12 neuronal damage [30]. In the present study, we found that the neuroprotective effect of DEX was closely related to the upregulated expression of SNHG16, while the neuroprotective effect of DEX on OGD-induced neuron injury was significantly decreased after knocking down SNHG16. Thus, DEX could attenuate ischemia-hypoxia-induced neurological damage by upregulating SNHG16.

In fact, as another type of non-coding RNA molecule, miRNAs also play an important role in ischemia-hypoxia-induced nerve damage. For example, miR-132 is upregulated in AD patients and promotes neuronal apoptosis by promoting Tau phosphorylation [31]. In the subarachnoid hemorrhage (SAH) model, glycine exerts neuroprotective effects through regulating the inflammatory response after SAH by regulating the miR-26b/PTEN signaling pathway [32]. In ischemic brain injury, miR-7a-5p can improve hypoxic-ischemic neuron damage by targeting α-synuclein (α-Syn), a protein that induces mitochondrial fragmentation, oxidative stress, and autophagy that promote neuronal cell death [33]. In the present study, we found that miR-10b-5p is upregulated in both MCAO and OGD-induced neuronal injury models, while DEX inhibits miR-10b-5p expression. Further overexpression and low expression of miR-10b-5p revealed that miR-10b-5p can promote neuronal apoptosis. Thus, DEX can exert neuroprotective effects by inhibiting the expression of miR-10b-5p.

In recent years, more and more studies have found that lncRNAs function as an endogenous competitive RNA by sponging specific miRNAs, and the miRNAs can bind to the 3′-UTR site of the targeted mRNA so as to regulate the transcription and translation of mRNA at the post-transcriptional level. This lncRNA-miRNA-mRNA regulatory network has been shown to play a key role in a variety of diseases. For example, SNHG16 enhances colon cancer cell growth through activating Akt pathway via competitively inhibiting miR-302a-3p [34]. In peripheral nerve injury, lncRNA-nuclear enriched abundant transcript 1 rich nuclear transcription factor 1 (NEAT1) promotes the proliferation and migration of Schwann cells by modulating the miR-34 a/SATB1 axis [35]. Similarly, lncRNA MIAT facilitate the expression of HMGB1 and regulates cerebral microvascular injury (CMEC) by competitively binding miR-204-5p [36]. In the beginning of this study, we found that after MCAO or OGD, the expression of SNHG16 and BDNF was upregulated, while the expression of miR-10b-5p was downregulated. Therefore, we were curious whether DEX could play a neuroprotective role by regulating SNHG16-miR-10b-5p-BDNF axis. Interestingly, our data demonstrated that SNHG16 sponged miR-10b-5p as a ceRNA and miR-10b-5p targeted BDNF. Hence, SNHG16 can promote the expression of BDNF by inhibiting miR-10b-5p, and the upregulated BDNF can help alleviate the injury of neurons after ischemia and hypoxia.

In summary, we demonstrate that DEX has significant neuroprotective effects on MCAO and OGD-mediated neurological damage. Mechanisticly, DEX exerts its effects by modulating the SNHG16-miR-10b-5p-BDNF axis (Fig. 6f). Overall, this study explored the neuroprotective effects and potential mechanisms of DEX in ischemia-anoxia-mediated brain injury, providing a new theoretical basis and method for the treatment of such a disease.

## Data Availability

The data used to support the findings of this study are available from the corresponding author upon request.
